# Lipoprotein(a) as a Cardiovascular Risk Factor: Clinical Significance and Therapeutic Perspectives

**DOI:** 10.3390/biomedicines14081743

**Published:** 2026-08-02

**Authors:** Michał Miszczak, Paweł Piwowarczyk, Grzegorz Sobieszek, Piotr Dobrowolski, Aleksander Prejbisz

**Affiliations:** 1 Department of Cardiology, 1st Military Clinical Hospital with the Outpatient Clinic, 20-049 Lublin, Poland; 2 Faculty of Medicine, Institute of Medical Sciences, The John Paul II Catholic University of Lublin, 20-708 Lublin, Poland; piwowarczyk.pawel@gmail.com; 3 Center for Cardiovascular Disease Prevention and Epidemiology, National Institute of Cardiology, 04-628 Warsaw, Poland

**Keywords:** lipoprotein(a), cardiovascular risk, residual cardiovascular risk, atherosclerosis, RNA therapeutics, risk stratification

## Abstract

Lipoprotein(a) (Lp(a)) is an elusive yet powerful cardiovascular risk factor, largely independent of LDL cholesterol. Elevated Lp(a) increases the risk of coronary artery disease, stroke, peripheral arterial disease, calcific aortic valve disease, heart failure, and abdominal aortic aneurysm. The challenge is that conventional lipid-lowering therapies have minimal impact on Lp(a), leaving patients with substantial residual risk. While observational and genetic studies generally support Lp(a) as a driver of cardiovascular events, results are not entirely consistent, and risk may differ between primary and secondary prevention. Novel therapies—including antisense oligonucleotides, siRNA therapeutics, CETP inhibitors, and experimental gene-editing approaches—show promising potential, reducing Lp(a) levels by up to 90%. Ongoing phase III trials will clarify whether these reductions translate into meaningful decreases in cardiovascular morbidity and mortality. This review critically examines the current evidence, highlights gaps in knowledge, and discusses emerging therapeutic strategies, emphasizing the need for individualized risk assessment. Lp(a) may soon shift from a “silent threat” to a precise, actionable target in cardiovascular prevention.

## 1. Introduction

Cardiovascular diseases (CVDs), including ischemic heart disease, stroke, and peripheral arterial disease, remain the leading cause of death worldwide [1]. Despite significant advances in low-density lipoprotein (LDL) cholesterol control, a substantial proportion of patients continue to experience cardiovascular events. Dyslipidemia remains one of the major modifiable risk factors, second only to hypertension [2]. Although LDL-C is the best-studied lipid marker, emerging evidence suggests that non-HDL cholesterol and apolipoprotein B (apoB) may better predict cardiovascular risk [3,4]. Non-HDL cholesterol reflects the full spectrum of circulating atherogenic lipoproteins, including LDL, VLDL, remnant cholesterol, and Lp(a), and is incorporated into the SCORE2 and SCORE2-OP algorithms for 10-year cardiovascular risk assessment in primary prevention. In the latest ESC/EAS recommendations, lipoprotein(a) is also recognized as an important cardiovascular risk modifier, and its measurement at least once during adulthood is recommended to improve individual cardiovascular risk stratification [5].

Lipoprotein(a) was first described in 1963 by the Norwegian researcher Kare Berg [6]. It consists of a lipid core—mainly phospholipids and cholesterol esters—combined with apolipoproteins apo(a) and apoB100. Lp(a) represents a significant residual atherosclerotic risk across multiple vascular beds and contributes to the pathogenesis of calcific aortic valve disease (CAVD), abdominal aortic aneurysm, and heart failure (HF) [7] Elevated Lp(a) levels (>125 nmol/L or >50 mg/dL) affect approximately 20% of the population [8]. Plasma Lp(a) levels are largely genetically determined by LPA gene polymorphisms, while environmental factors account for only ~10%. Current Polish guidelines classify Lp(a) concentrations according to cardiovascular risk: moderate (30–50 mg/dL; 75–125 nmol/L), high (50–180 mg/dL; >125–450 nmol/L), and very high (>180 mg/dL; >450 nmol/L) [9].

**Figure 1:**
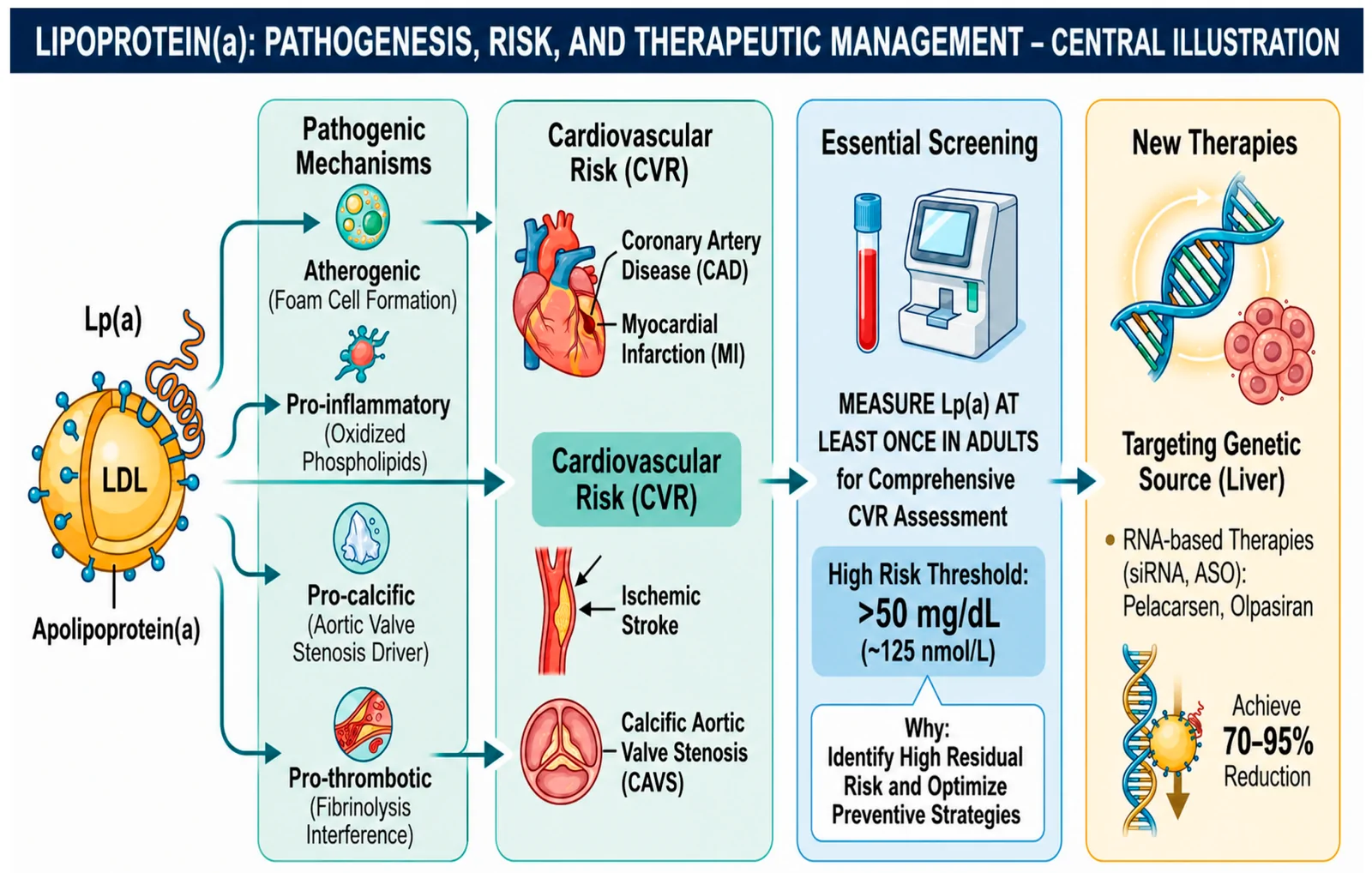
**Central Illustration.** Pathogenesis, cardiovascular risk, screening, and therapeutic management of lipoprotein(a). Original illustration created by the authors.

Although extensive epidemiological, genetic, and Mendelian randomization evidence supports Lp(a) as an independent causal cardiovascular risk factor, considerable heterogeneity remains regarding its prognostic value across different populations and clinical settings. The observed discrepancies likely reflect complex interactions between baseline LDL-C levels, concomitant lipid-lowering therapies, inflammatory burden, genetic variability of the LPA gene and apo(a) isoform size, as well as differences in laboratory assays and reporting units for Lp(a) [10,11]. Addressing these sources of heterogeneity is essential for improving cardiovascular risk stratification and implementing precision medicine approaches in patients with elevated Lp(a). This review critically examines the current understanding of Lp(a), highlighting both its pathogenic role and the existing areas of uncertainty. The Central Illustration (Figure 1) provides an overview of the structural features of Lp(a), its pathogenic mechanisms, clinical manifestations, and emerging therapeutic approaches discussed throughout this review.

## 2. Structural and Molecular Features of Lp(a)

**Figure 2:**
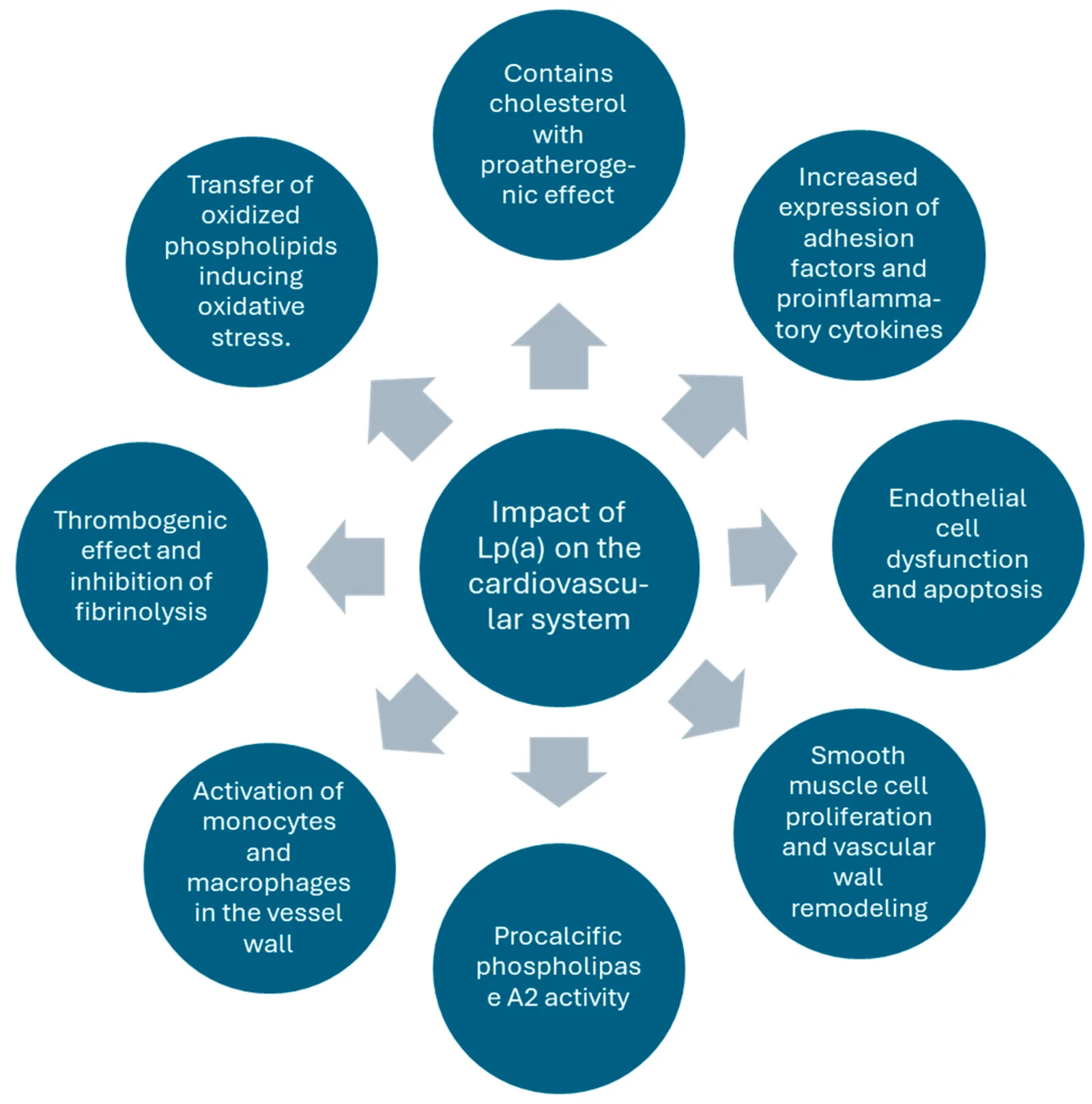
Role of Lp(a) in the pathogenesis of heart diseases.

Lp(a) is an LDL-like lipoprotein particle composed of a low-density lipoprotein (LDL) molecule covalently linked to apo(a), a liver-synthesized glycoprotein structurally homologous to plasminogen. Although apo(a) exhibits marked structural homology with plasminogen and experimental studies suggest inhibition of fibrinolysis through competition with plasminogen binding, convincing clinical evidence demonstrating an independent prothrombotic effect of Lp(a) remains limited. Apo(a) consists of a single kringle V (KV) domain and ten kringle IV (KIV1–KIV10) subtypes. The number of KIV2 repeats (ranging from 3 to >50), determined by the LPA gene, influences apo(a) molecular weight and circulating Lp(a) levels; fewer repeats correspond to higher plasma concentrations. Apo(a) is covalently attached to apolipoprotein B100 of the LDL particle through a single disulfide bond involving the kringle IV type 9 (KIV-9) domain [12]. Oxidized phospholipids (OxPLs) associated with KIV apo(a) and apoB100 contribute to calcific aortic valve disease (CAVD) pathogenesis [13]. Lp(a) also carries bioactive enzymes, including lipoprotein-associated phospholipase A2 (Lp-PLA2) and autotaxin [14], which promote local inflammation and osteogenic differentiation of valvular interstitial cells, thereby contributing to valvular calcification. The principal pathogenic mechanisms of Lp(a) are summarized in Figure 2.

## 3. Clinical Impact of Lipoprotein(a) Across Vascular Beds

### 3.1. Is Lp(a) a Uniform Risk Factor Across Vascular Beds? One Molecule—Different Faces of Risk

Accumulating evidence suggests that Lp(a) is not a uniform cardiovascular risk factor and that its impact varies across vascular territories, as well as between primary and secondary prevention settings. These differences are discussed in greater detail in the following section (Section 3.2), together with the available clinical evidence. In individuals with very high Lp(a) concentrations (>450 nmol/L), the overall cardiovascular risk is comparable to that observed in patients with heterozygous familial hypercholesterolemia [5].

In the largest observational study to date, including 460,500 participants from the UK Biobank, a linear increase in ASCVD risk of 11% was observed for every 50 mg/dL increment in Lp(a) levels. Importantly, subgroup analyses revealed substantial heterogeneity: high Lp(a) concentrations (>150 mg/dL) were associated with a 50% increase in cardiovascular risk among individuals without established ASCVD, but only a 16% increase among those with pre-existing disease [15]. These findings suggest that the prognostic relevance of Lp(a) may be modified by disease stage and the intensity of background preventive therapies.

### 3.2. Coronary Artery Disease—A Strong Signal, but Not Uniform Across Patients

In a prospective study of 267 patients undergoing coronary computed tomography angiography, individuals with Lp(a) levels > 125 nmol/L exhibited a significantly greater atherosclerotic plaque burden compared with those with lower concentrations (6.9% vs. 3.0%; *p* = 0.01). Moreover, higher Lp(a) levels were associated with a greater prevalence of low-attenuation plaques, which are considered more vulnerable to rupture, as well as with increased pericoronary adipose tissue inflammation [16]. These observations suggest that Lp(a) may influence not only plaque quantity but also plaque composition and stability.

Berman et al. demonstrated an almost linear increase in cardiovascular event risk with rising Lp(a) levels in individuals without established ASCVD, with a 93% higher risk observed in those with Lp(a) concentrations >216 nmol/L compared with <42 nmol/L [17]. In contrast, among patients with established ASCVD, the relationship was non-linear, with a 21% increase in major adverse cardiovascular events (MACE) for Lp(a) levels of 112–215 nmol/L and a 26% increase for levels >216 nmol/L. These data support the concept that risk thresholds for Lp(a) should differ between primary and secondary prevention.

A meta-analysis of 11 studies comprising 562,301 participants confirmed a significant association between elevated Lp(a) levels and cardiovascular events in both primary and secondary prevention populations, independent of high-sensitivity C-reactive protein (hs-CRP). However, the magnitude of the effect varied considerably, suggesting interactions between Lp(a), inflammatory pathways, and other cardiovascular risk factors [18].

Collectively, these findings suggest that the particularly strong association between Lp(a) and coronary artery disease likely reflects the combined proatherogenic, proinflammatory, and prothrombotic properties of Lp(a). Oxidized phospholipids carried by apo(a) promote endothelial dysfunction, monocyte recruitment, foam-cell formation, and plaque vulnerability, providing biological plausibility for the consistent epidemiological associations.

### 3.3. Myocardial Infarction—The Role of Extreme Levels and Sex-Specific Effects

Data from the Copenhagen studies indicate a strong association between very high Lp(a) concentrations and myocardial infarction (MI) risk, with individuals above the 99th percentile exhibiting more than a twofold higher risk of MI than those with low Lp(a) levels [19]. Elevated Lp(a) concentrations have also been associated with higher SYNTAX scores, suggesting more extensive and complex coronary artery disease [20].

However, the prognostic impact of Lp(a) appears less consistent in secondary prevention. While a Slovenian cohort demonstrated an association between elevated Lp(a) and recurrent MI exclusively in women older than 65 years, potentially reflecting postmenopausal hormonal changes [21], several other studies failed to confirm a consistent relationship between Lp(a) and recurrent cardiovascular events [22,23].

These discrepancies may be explained by the widespread use of intensive lipid-lowering therapy, lower achieved LDL-C levels, and competing cardiovascular risk factors in secondary prevention populations, all of which may attenuate the relative contribution of Lp(a) to recurrent events.

### 3.4. Aortic Valve Disease—The Most Consistent and Causal Association

Among all vascular territories, the relationship between Lp(a) and calcific aortic valve disease appears the most robust and consistent. A meta-analysis including 52,931 participants demonstrated a 76% increased risk of CAVD among individuals with Lp(a) levels >50 mg/dL [24]. Elevated Lp(a) has also been shown to accelerate disease progression, as reflected by faster increases in mean transvalvular gradients [25].

In patients with established CAVD, higher Lp(a) concentrations were associated with worse clinical outcomes, including an increased need for aortic valve replacement and higher all-cause mortality [26]. Similar associations were observed for oxidized phospholipids bound to apolipoprotein B100, further supporting a direct pathogenic role of Lp(a) in valvular calcification.

The remarkable consistency of these findings distinguishes CAVD from other cardiovascular manifestations associated with Lp(a). Strong epidemiological evidence, together with genetic and experimental studies, indicates that oxidized phospholipids transported by Lp(a) promote valvular inflammation, osteogenic differentiation of valvular interstitial cells, and progressive leaflet calcification, supporting a direct causal role of Lp(a) in disease initiation and progression.

### 3.5. Stroke—A Weaker and More Heterogeneous Association

The association between Lp(a) and stroke appears less pronounced than that observed for coronary artery disease. An approximate 10–13% increase in ischemic stroke risk has been reported per one standard deviation increase in Lp(a) levels [27,28], although higher risk estimates have been observed in case–control studies compared with prospective cohorts [29].

Elevated Lp(a) concentrations have also been linked to a higher prevalence of large-artery cerebral atherosclerosis and hemorrhagic stroke [30]. Recent meta-analytic data including 11,958 patients with ischemic stroke demonstrated poorer functional outcomes, as assessed by the modified Rankin Scale, among individuals with elevated Lp(a), particularly during the early post-stroke period. Notably, this effect diminished substantially by 12 months, raising questions regarding the long-term prognostic relevance of Lp(a) in stroke recovery [31].

The inconsistent association between Lp(a) and stroke probably reflects differences in the underlying biology of cerebrovascular disease. Unlike coronary artery disease, ischemic stroke represents a heterogeneous clinical syndrome in which only the large-artery atherosclerotic subtype is expected to be directly influenced by the proatherogenic effects of Lp(a).

### 3.6. Peripheral Artery Disease and Heart Failure—Direct or Indirect Risk?

The association between Lp(a) and peripheral artery disease (PAD) as well as abdominal aortic aneurysm is strong and consistent, particularly at extreme Lp(a) concentrations [32,33]. Emerging data also suggest a potential link with aortic dissection, although these findings require further validation [34].

In the context of heart failure (HF), the impact of Lp(a) appears to be largely mediated through coronary artery disease and calcific aortic valve disease. Nevertheless, Mendelian randomization analyses support a modest but statistically significant association between elevated Lp(a) levels and HF risk, indicating that Lp(a) may represent more than a simple risk marker [35,36].

The differing strength of the association between Lp(a) and PAD versus HF likely reflects distinct underlying disease mechanisms. While PAD is predominantly driven by systemic atherosclerosis, a process strongly influenced by the proatherogenic and proinflammatory properties of Lp(a), heart failure represents a heterogeneous clinical syndrome in which the effects of Lp(a) are largely indirect, mediated through coronary artery disease and calcific aortic valve disease. These differences emphasize that the clinical impact of Lp(a) depends on the predominant pathogenic mechanisms underlying each disease rather than on elevated Lp(a) concentrations alone.

Taken together, these findings indicate that the prognostic significance of Lp(a) is highly context-dependent and influenced by disease-specific pathophysiological mechanisms, background lipid-lowering therapy, and the clinical setting. Notably, the relative contribution of Lp(a) to cardiovascular risk appears to differ between primary and secondary prevention, being more pronounced in individuals without established ASCVD than in those receiving contemporary secondary prevention therapies. Therefore, elevated Lp(a) should be interpreted as a dynamic cardiovascular risk modifier rather than a uniform predictor of risk across all cardiovascular diseases.

## 4. Unresolved Questions and Clinical Controversies

The highly unique and complex structure of Lp(a) presents challenges in standardizing laboratory assays. The variability in the number of KIV-2 repeats and the frequent codominant expression of two apo(a) isoforms of different molecular size, encoded by the two *LPA* alleles, complicate assay calibration and hamper comparisons between different laboratory methods.

The available literature overwhelmingly supports the role of Lp(a) as a cardiovascular risk factor. However, several studies have questioned this role. In one study, among patients following a recent ACS (acute coronary syndrome), Lp(a) concentration was not associated with an increased incidence of cardiovascular events [31]. In another study, post-STEMI patients exhibited an increased risk of recurrent myocardial infarction only in the subgroup with extremely elevated Lp(a) (>135 mg/dL), while Lp(a) levels did not correlate with increased mortality [37]. Other authors have questioned the impact of Lp(a) on cardiovascular risk among patients with established ASCVD and an average LDL-cholesterol level below 130 mg/dL [38]. These findings underscore the need for further studies to address existing evidence gaps, particularly in secondary prevention populations.

The role of Lp(a) in the pathogenesis of type 2 diabetes remains debated. Several studies report an increased risk of developing type 2 diabetes in patients with low Lp(a) levels [39]. Paradoxically, the incidence of microangiopathic complications, particularly neuropathy and retinopathy, appears higher in patients with elevated Lp(a) levels [40]. A population-based study including 563,420 patients using Mendelian randomization, however, found no association between Lp(a) levels and the risk of developing diabetes [41]. Despite strong observational evidence, significant gaps remain regarding risk thresholds, target populations, and real-world clinical benefits.

## 5. Emerging Lp(a)-Lowering Therapies: Testing the Causality Hypothesis

Currently, the primary target of lipid-lowering therapy, particularly in patients with ASCVD, is LDL-C. Statins remain the cornerstone of LDL-C–lowering therapy but have no clinically meaningful Lp(a)-lowering effect. A patient-level meta-analysis of six randomized clinical trials demonstrated that statin therapy was associated with a modest increase in Lp(a) concentrations, ranging from 8.5% to 19.6% [42]. Similarly, an analysis of the JUPITER trial showed a small but statistically significant increase in Lp(a) concentrations during rosuvastatin therapy, while confirming that elevated Lp(a) remained associated with residual cardiovascular risk despite statin treatment [43]. The magnitude of the statin-associated increase may depend on apo(a) isoform size, with individuals carrying fewer KIV-2 repeats, and therefore smaller apo(a) isoforms, exhibiting a greater increase in Lp(a) concentrations during statin therapy. However, the clinical relevance of this effect remains uncertain and is unlikely to influence therapeutic decisions, given the well-established cardiovascular benefits of statins. Furthermore, a network meta-analysis of 20 studies including 23,605 participants did not demonstrate a statistically significant overall effect of statins on Lp(a) concentrations, although a non-significant trend toward Lp(a) reduction was observed with pitavastatin [44].

Bempedoic acid and ezetimibe have no significant impact on Lp(a) concentrations. The only currently available pharmacologic agents shown to reduce Lp(a) levels are PCSK9 inhibitors and inclisiran, a small interfering RNA (siRNA) targeting hepatic PCSK9 synthesis. Lomitapide, a microsomal triglyceride transfer protein (MTP) inhibitor [45], and evinacumab, a monoclonal antibody targeting angiopoietin-like protein 3 (ANGPTL3) [46], have also been reported to reduce Lp(a) concentrations, particularly in patients with homozygous familial hypercholesterolemia. However, their effects on Lp(a) are modest and these agents are not used specifically for Lp(a)-lowering therapy [47].

In the FOURIER trial, evolocumab reduced Lp(a) levels by approximately 27% compared with placebo. Notably, evolocumab more effectively reduced the risk of coronary events in patients with higher baseline Lp(a) compared with those at or below the median (23% versus 7%) [48]. This suggests that the overall reduction in MACE risk in the study population may have been partly attributable to the decrease in Lp(a) levels. Alirocumab reduces Lp(a) levels by approximately 23–27% depending on dose, with an average reduction of about 23.5% across phase III studies [49]. Analysis of the ODYSSEY OUTCOMES trial demonstrated that alirocumab decreased the risk of PAD-related events by 31% (95% CI, 0.54–0.89; *p* = 0.004) [50]. Inclisiran exhibits a smaller potential for Lp(a) reduction compared to PCSK9 inhibitors. In the ORION-10 (ASCVD patients) and ORION-11 (ASCVD or ASCVD-risk patients) trials, median Lp(a) reductions were 21.9% and 18.9%, respectively [51].

Interesting insights regarding the potential modification of Lp(a)-dependent cardiovascular risk in individuals without established ASCVD come from the NHANES III study. Over 26 years of follow-up, regular use of acetylsalicylic acid (ASA) in participants with elevated Lp(a) (>50 mg/dL) was associated with a 52% reduction in mortality in multivariable modeling (HR = 0.48, 95% CI: 0.28–0.83). No such effect was observed among participants with normal Lp(a) levels [52]. These striking findings may provide a rationale for future recommendations on primary prevention of ASCVD in individuals with hyperlipoproteinemia(a).

A highly effective and currently available method for lowering elevated Lp(a) in patients with progressive ASCVD is apheresis. A systematic review of 10 studies on Lp(a) apheresis confirmed its high efficacy in achieving long-term reductions in Lp(a) levels (53–73%), which was associated with substantial reductions in cardiovascular events (54–90%) [53].

A breakthrough in the treatment of hyperlipoproteinemia(a) may come from several emerging therapeutic strategies targeting Lp(a). The characteristics of the most advanced agents currently under clinical investigation are summarized in Table 1. These therapies can be broadly classified into four groups:

- Antisense oligonucleotides (ASO)—pelacarsen
- Small interfering RNA (siRNA)—olpasiran, zerlasiran, lepodisiran
- Lp(a) inhibitors—muvalalpin
- Cholesteryl ester transfer protein (CETP) inhibitors—obicetrapib

Pelacarsen is a second-generation antisense oligonucleotide administered subcutaneously once monthly, targeting LPA mRNA in hepatocytes. Phase I and II trials demonstrated high efficacy, with reductions in Lp(a) levels ranging from 72% to 92%, alongside a favorable safety profile [54]. The ongoing phase III Lp(a)HORIZON trial (NCT04023552) compares pelacarsen 80 mg versus placebo in patients with clinically manifest atherosclerotic ASCVD and Lp(a) levels above 70 mg/dL. This will be the first completed phase III trial to determine whether lowering Lp(a) concentrations translates into a reduction in cardiovascular events and mortality. Study completion is expected in June 2026 [55].

Olpasiran is an N-acetylgalactosamine-conjugated small interfering RNA (siRNA) administered subcutaneously every 12 weeks. Its mechanism of action involves inhibiting the translation of mRNA encoding apolipoprotein(a), thereby reducing its synthesis in hepatocytes. In the extension follow-up of the phase II OCEAN(a)-DOSE trial, participants receiving olpasiran 75 mg every 12 weeks or 225 mg every 12 weeks maintained placebo-adjusted reductions in Lp(a) of 76.2% and 84.4%, respectively, at week 60 after treatment initiation. Remarkably, Lp(a) reduction persisted by 40–50% nearly 12 months after the final dose [56]. The ongoing phase III OCEAN(a) trial (NCT05581303) is evaluating the effect of olpasiran on cardiovascular outcomes and mortality in patients with atherosclerotic ASCVD and Lp(a) levels above 200 nmol/L. Study completion is anticipated in March 2028 [57].

Lepodisiran, another siRNA-based therapeutic, demonstrated Lp(a) reductions exceeding 95% in phase I and II studies. The ongoing phase III ACCLAIM-Lp(a) trial (NCT06292013) is unique in that it evaluates the drug’s efficacy both in patients with established ASCVD and in individuals without ASCVD but with cardiovascular risk factors. Completion is expected in March 2029.

Obicetrapib, a cholesteryl ester transfer protein (CETP) inhibitor, exerts a broad-spectrum lipid-modifying effect by reducing levels of atherogenic TC, LDL-C, non-HDL-C, Lp(a), and apoB100. Phase II trials demonstrated Lp(a) reductions of 33.8% and 56.5% compared with placebo, depending on the dose [58]. The phase III BROADWAY trial (NCT05142722), completed in September 2024, enrolled 2532 patients with ASCVD and/or HeFH who received obicetrapib 10 mg or placebo. According to preliminary conference data (unpublished), the study achieved a 21% reduction in the primary composite endpoint (all-cause mortality, nonfatal myocardial infarction, nonfatal stroke, and coronary revascularization) in favor of obicetrapib. As a CETP inhibitor, obicetrapib also markedly increases HDL-cholesterol and apolipoprotein A-I concentrations [58]. However, whether these changes contribute independently to its potential cardiovascular benefits remains uncertain, as reductions in apoB-containing lipoproteins are generally considered the principal mechanism underlying cardiovascular risk reduction.

Other agents specifically targeting Lp(a), including zerlasiran and muvalaplin, remain in earlier stages of clinical development.

Gene therapy approaches based on CRISPR–Cas9 technology are also being explored [59]. The investigational molecule CTX320, which contains Cas9 mRNA, is administered as a single dose and has been shown to edit approximately 70% of the LPA gene, resulting in a 90% reduction in Lp(a) levels.

### Future Perspectives: Towards Precision Medicine in Lipoprotein(a) Management

Despite remarkable advances in the development of Lp(a)-lowering therapies, several important challenges must be addressed before these agents can be fully integrated into routine clinical practice. Future management of hyperlipoproteinemia(a) will likely extend beyond simply reducing circulating Lp(a) concentrations and move toward a precision medicine approach that incorporates individual cardiovascular risk, disease phenotype, genetic background, and expected treatment benefit.

One of the key unresolved issues is the identification of patients who are most likely to benefit from Lp(a)-targeted therapies. Although current evidence consistently supports elevated Lp(a) as an independent causal risk factor for ASCVD and calcific aortic valve disease, it remains uncertain whether pharmacological treatment should be offered to all individuals with elevated Lp(a) concentrations or preferentially to selected high-risk populations, such as patients with premature ASCVD, progressive disease despite optimal lipid-lowering therapy, recurrent cardiovascular events, or markedly elevated Lp(a) concentrations. The results of ongoing cardiovascular outcome trials will be crucial in defining these treatment strategies.

Another priority is the standardization of Lp(a) measurement and interpretation. Despite growing consensus regarding the need for one-time lifetime measurement of Lp(a), considerable variability persists between laboratory assays because of apo(a) isoform heterogeneity and differences in reporting units (mg/dL versus nmol/L). Harmonization of analytical methods and the establishment of universally accepted clinical thresholds will be essential to facilitate widespread implementation of Lp(a)-guided risk assessment and therapeutic decision-making.

The future role of Lp(a) is also expected to evolve within the broader framework of personalized cardiovascular prevention. Rather than considering Lp(a) as an isolated biomarker, its clinical significance should be interpreted together with traditional cardiovascular risk factors, LDL-C burden, inflammatory status, family history, imaging findings, and genetic predisposition. Such an integrated approach may improve identification of individuals with substantial residual cardiovascular risk despite optimal management of conventional risk factors.

Ultimately, the ongoing phase III outcome trials evaluating pelacarsen, olpasiran, lepodisiran, and other novel therapies will determine whether selective Lp(a) reduction translates into meaningful reductions in cardiovascular morbidity and mortality. Positive results would establish Lp(a) as the first genetically determined lipid parameter for which targeted pharmacological lowering confers proven clinical benefit, fundamentally changing current approaches to cardiovascular risk assessment and prevention.

## 6. When Lifestyle Is Not Enough: Facing the Lp(a) Challenge

What do we do when a risk factor lurks in the background, largely untouchable by the drugs we rely on most? Elevated Lp(a) is exactly that: a stealthy culprit contributing to cardiovascular events even when LDL-C is controlled. Clinicians and patients cannot simply wait for new therapies; action is still possible. Long-term data from the EPIC–Norfolk study highlight that intensive modification of traditional cardiovascular risk factors can significantly reduce events, even in individuals with Lp(a) above 50 mg/dL [60]. Among modifiable risk factors, smoking cessation deserves particular emphasis, as smoking may further increase cardiovascular risk in individuals with elevated Lp(a). Yet here is the caveat: these interventions—weight control, exercise, smoking cessation, blood pressure and glucose management—do not lower Lp(a) itself. Lifestyle modification is crucial but the residual risk remains. The American Heart Association’s “ideal cardiovascular health” metrics offer a compelling framework, showing up to 75% reduction in cardiovascular risk among those with high Lp(a). The data suggest that some patients may benefit less, others more, and we still do not know exactly who crosses the threshold where lifestyle alone is insufficient. This is where clinical judgment becomes critical. We must individualize risk stratification, recognize the persistent threat of elevated Lp(a), and prepare for the era of Lp(a)-specific therapies. Until then, lifestyle is necessary—but it is not sufficient. The real question remains: will upcoming pharmacologic interventions finally tip the balance and convert high Lp(a) from a silent hazard into a manageable risk?

## 7. Conclusions

Lp(a): From Risk Marker to Therapeutic Target

Lipoprotein(a) is a potent, genetically determined, and underutilized causal marker of cardiovascular risk, driving atherogenesis, inflammation, and thrombosis beyond what standard LDL-C models capture. Although targeted Lp(a)-lowering therapies are still in clinical trials, establishing its baseline level is critical today. It accurately identifies individuals at the highest residual risk who will become prime candidates for future drugs, while immediately signaling the need for stringent control of other risk factors and aggressive lifestyle modifications.

In Poland, this proactive stratification is increasingly supported by public health frameworks. The widespread availability of free Lp(a) screening for young people within the “Moje Zdrowie” initiative ensures early detection, while the “10 for Health” (*10 dla Zdrowia*) program provides a practical tool to optimize prevention across ten risk domains, seven of which focus directly on lifestyle. Ultimately, routine Lp(a) measurement bridges the gap between identifying future therapeutic candidates and enforcing rigorous, multi-factorial prevention strategies in contemporary practice.

## Figures and Tables

**Table 1:** Drugs Under Clinical Investigation for Lowering Lipoprotein(a) Levels—Summary.

Drug and Mechanism of Action	Degree of Lp(a) Reduction	Dosage and Administration Route	Clinical Trial Phase/Completion Date	Most Common Adverse Events
Pelacarsen (ASO targeting LPA mRNA)	60–80%	Subcutaneous, every 4 weeks	Phase III—Lp(a) HORIZON Estimated completion: Jun 2026	Injection site reactions
Olpasiran (siRNA targeting LPA mRNA)	70–95%	Subcutaneous, every 12 weeks	Phase III—OCEAN(a) Estimated completion: Mar 2028	Injection site reactions
Lepodisiran (siRNA targeting LPA mRNA)	90–94%	Subcutaneous, twice (Day 0 and 180)	Phase III—ACCLAIM-Lp(a) Estimated completion: 2029	Injection site reactions, transient elevations of AST/ALT, headache
Zerlasiran (siRNA targeting LPA mRNA)	80–95%	Subcutaneous, every 16–24 weeks	Phase II completed (ALPACAR-360, 2024) Phase III planned	Injection site reactions
Muvalaplin (apo(a) synthesis inhibitor)	65–86%	Oral, daily	Phase II completed (KRAKEN) Phase III—MOVE-Lp(a) initiated Sept 2025	Mild and transient (headache, back pain, nausea, diarrhea, fatigue)
Obicetrapib (CETP inhibitor)	33–57%	Oral, daily	Phase III trials completed: BROADWAY (ASCVD/HeFH) and BROOKLYN (HeFH) Ongoing: PREVAIL (CV outcomes)	Headache, respiratory infections, nausea

## Data Availability

No new data were created or analyzed in this study. Data sharing is not applicable to this article.
